# Identification of a Novel hsa_circ_0058058/miR-324-5p Axis and Prognostic/Predictive Molecules for Acute Myeloid Leukemia Outcome by Bioinformatics-Based Analysis

**DOI:** 10.3390/biology13070487

**Published:** 2024-06-30

**Authors:** Sema Misir, Serap Ozer Yaman, Nina Petrović, Ahmad Šami, Osman Akidan, Ceylan Hepokur, Yuksel Aliyazicioglu

**Affiliations:** 1 Department of Biochemistry, Faculty of Pharmacy, Sivas Cumhuriyet University, 58140 Sivas, Turkey; cozsoya@gmail.com; 2 Department of Medical Biochemistry, Faculty of Medicine, Karadeniz Technical University, 61080 Trabzon, Turkey; serapozer@ktu.edu.tr (S.O.Y.); yukselayazici@hotmail.com (Y.A.); 3 Department of Medical Biochemistry, Trabzon Faculty of Medicine, University of Health Sciences, 61080 Trabzon, Turkey; 4 Laboratory for Radiobiology and Molecular Genetics, Department of Health and Environment, “VINČA” Institute of Nuclear Sciences-National Institute of the Republic of Serbia, University of Belgrade, 11351 Belgrade, Serbia; dragoninspiration@yahoo.com; 5 Department for Experimental Oncology, Institute for Oncology and Radiology of Serbia, 11351 Belgrade, Serbia; 6 Cellular and Molecular Radiation Oncology Laboratory, Department of Radiation Oncology, Universitaetsmedizin Mannheim, Medical Faculty Mannheim, Heidelberg University, 68167 Mannheim, Germany; ahmad.sami@medma.uni-heidelberg.de; 7 Department of Hematology, Mengücek Gazi Education and Research Hospital, 24100 Erzincan, Turkey; osmanakidandr@hotmail.com

**Keywords:** acute myeloid leukemia, circRNA, non-coding RNAs, miRNA

## Abstract

**Simple Summary:**

Acute myeloid leukemia (LAML) is among the most common types of hematological cancer. In recent years, it has been shown that circular RNA (circRNA) and microRNA (miRNA) can be used as markers for diagnosis and treatment results in tumor development, in the early stages of the tumor or after chemotherapy. This study aimed to identify the disease-related circRNA–miRNA–mRNA network by bioinformatic analysis and investigate the mechanisms in the development and progression of LAML. Bioinformatics analyses identified the hsa_circ_0058058/miR-324-5p axis in LAML and its possible functions in LAML development. It was found that hsa_circ_0058058 could regulate the expression of AP1G1 and SP1 through miR-324-5p to promote angiogenesis, cell cycle, and DNA replication processes. Downregulation of Hsa circ-0058058 may contribute to the anticancer functions of miR-324-5p on LAML tumorigenesis. Upregulation of miR-324-5p could abolish the oncogenic effects of AP1G1 and SP1 on LAML tumorigenesis.These analyses demonstrated their important role by showing that these molecules can be used as a diagnostic biomarker and therapeutic target for the treatment of LAML.

**Abstract:**

Acute myeloid leukemia (LAML) is one of the most prevalent hematological malignancies. In recent years, while targeted approaches have shown promise in the fight against cancer, the treatability and prognosis of patients remain inadequate due to the shortage of drugs. Noncoding RNAs, especially circular RNA (circRNA) and microRNA (miRNA), have been shown to play a unique role in tumor development. This study aims to identify the disease-associated circRNA–miRNA–mRNA network by bioinformatic analysis and investigate the mechanisms in the development and progression of LAML. Additionally, it reveals the promising roles of these molecules as a diagnostic biomarker and therapeutic target for LAML treatment. Using various bioinformatics approaches, we identified the hsa_circ_0058058/miR-324-5p axis in LAML and its possible functions in LAML development. According to our results, hsa circ-0058058 can regulate the expression of AP1G1 and SP1 through miR-324-5p to support angiogenesis, the cell cycle, and DNA replication processes. Downregulation of hsa circ-0058058 may contribute to the anticancer functions of miR-324-5p on LAML tumorigenesis, and upregulation of miR-324-5p can abolish the oncogenic effects of AP1G1 and SP1 on LAML tumorigenesis. Additionally, highly enriched pathways indicated possible interactions between molecules underlying LAML pathology. Targeted molecules within this network may be able to function as therapeutic and diagnostic biomarkers for disease, while more research and clinical confirmation are needed.

## 1. Introduction

Acute myeloid leukemia (LAML) is a heterogeneous and invasive hematological malignancy characterized by the differentiation and acquisition of cytogenetic and molecular abnormalities of hematopoietic stem cells [1,2]. With the development of chemotherapy, immunotherapy, targeted medicines, and bone marrow transplantation, LAML has come to be recognized as a curable malignancy, but the death rate among LAML patients is still very high [3,4]. Despite advances in treatment, more than 50% of LAML patients relapse and develop resistance to chemotherapy [1,2]. Therefore, understanding the molecular mechanisms underlying the progression of LAML is important for developing effective treatment strategies and identifying innovative diagnostic and prognostic molecular biomarkers.

According to current research, non-coding RNAs, including microRNAs (miRNAs) and circular RNAs (circRNAs), are involved in many disease processes as well as a variety of biological activities [5]. circRNAs are non-coding RNA molecules that form covalently closed loops that do not contain 5′ caps and 3′ poly (A) tails [6]. circRNAs have been shown to have biological roles as miRNA sponges, transcriptional or post-transcriptional regulators of gene expression levels, regulators of gene expression, and RBP (RNA-Binding Protein) sponges [7]. Studies have shown that the expression levels of circRNAs vary in different types of cancer and that the changing expression of circRNAs plays a vital role in tumor initiation and progression [8]. In particular, it has been reported that circRNAs can enhance or suppress the development of cancer genes associated with migration, differentiation, proliferation, and carcinogenesis by repressing miRNA species [9]. Research findings indicate that circRNAs contribute significantly to the advancement of LAML via many pathways [1]. However, the specific underlying molecular mechanisms of most circRNAs involved in LAML pathogenesis still remain unclear.

miRNAs are small non-coding RNA molecules with a length of 18–25 nucleotides. miRNAs are involved in post-transcriptional regulation of gene expression, causing repression of protein synthesis or mRNA degradation [10]. Through the post-transcriptional regulation of target genes, they regulate numerous biological processes, including cell proliferation, differentiation, and tumor development and progression [11]. Deregulation of miRNA–mRNA interaction causes many diseases, including cancer. miRNAs, as genome elements that do not encode form proteins, play an important role in cancer initiation, progression, invasion, and metastasis and tumorigenesis control at every stage [12]. Therefore, miRNAs are good targets for developing new therapeutic molecules and offer significant promising anticancer strategies for pre-metastasis treatment [13]. In addition, increasing evidence suggests that miRNAs and circRNAs can be used as markers for the diagnosis and treatment outcome of LAML at early stages or after chemotherapy [11,14].

In recent years, bioinformatic analysis has been frequently used to examine the progression of cancer and identify possible potential therapeutic targets. Due to the advantages of these analyses, such as different sample sizes and microarray and sequencing platforms, they can overcome the inconsistent results that appear in the literature [15].

This study used available microarray and sequencing data to identify the disease-associated circRNA–miRNA–mRNA network. This study aims to explain the potential function and regulatory mechanisms of the hsa_circ_0058058/miR-324-5p regulatory network in the pathogenesis or clinical prognosis of LAML. Additionally, it reveals the promising roles of these molecules as diagnostic biomarkers and therapeutic targets for LAML treatment.

## 2. Methods

### 2.1. Identification of circRNAs in LAML

The circRNA data of LAML were obtained from the CircR2Disease database. CircR2Disease provides experimentally supported data between circRNAs and diseases. Six circRNAs with an experimentally proven association with LAML were identified using the CircR2Disease database (http://bioinfo.snnu.edu.cn/CircR2Disease/article/Browse.aspx) (accessed on 26 September 2023) [16].

### 2.2. Identification of circRNAs-Mediated miRNA in LAML

For the detection of “hub” miRNAs, common targets for circRNAs differentially expressed in LAML miRNA, miRNET (https://www.mirnet.ca/, accessed on 7 May 2024), an online platform, was used [17]. In order to determine common miRNAs potentially associated with hsa_circ_0075001 (NPM1 circRNA), hsa_circ_0004277 (WDR37 circRNA), hsa_circ_0035381 (PIGB circRNA), hsa_circ_0004136 (KCNQ5), hsa_circ_0058058 (ATIC), and hsa_circ_0017446 (WDR37 circRNA), “Multiple query types” was checked, and H. sapiens (human) organism was specified. In “Multiple query”, circRNA and disease items were chosen. The list of 5 circRNAs was added under the “Official gene symbol” ID type, and Myeloid, Leukemia, Acute was selected under the “Diseases” tab. The interaction between circATIC (hsa_circ_0058058) and hsa-miR-324-5p was predicted by Circular RNA Interactome software (https://circinteractome.nia.nih.gov, accessed on 7 May 2024) [18].

### 2.3. Prediction of miRNA Targets

We employed the miRNET database (https://www.mirnet.ca/, accessed on 7 May 2024) [17] to determine the potential target gene of hsa-miR-324-5p. Additionally, for HL-60, Kasumi 6, LAML-2, and KG-1 cells, the online database miRDB (https://mirdb.org/, accessed on 7 May 2024) was used to estimate the targets of hsa-miR-324-5p. The common target genes obtained from miRNET and miRDB were determined [19].

### 2.4. Verification of Gene Expression in LAML Cohort I

TIMER2 (tumor immune estimation resource, version 2, http://timer.cistrome.org/, accessed on 7 May 2024) in the ‘Gene DE’ module, GEPIA2 (Gene Expression Profiling Interactive Analysis, version 2) l (http://gepia2.cancer-pku.cn/#analysis, accessed on 7 May 2024), and UALCAN tools (http://ualcan.path.uab.edu/, accessed on 7 May 2024) were used to analyze the gene expression of Adapter-associated protein complex 1 gamma 1 (AP1G1) and Specificity protein 1 (SP1) in LAML. TIMER offers a variety of modules that let users interactively study connections between immune infiltrates and a variety of variables, such as gene expression, clinical outcomes, somatic mutations, and somatic copy number variations [20,21]. Based on information from the Cancer Genome Atlas (TCGA) and Genotype-Tissue Expression (GTEx), the interactive web program Gene Expression Profiling Interactive Analysis (GEPIA) provides a variety of visualization and analysis capabilities for gene expression. Using the gene expression profiling database GEPIA (http://gepia.cancer-pku.cn/, accessed on 7 May 2024), we identified changes in the expression of identified genes between LAML and healthy tissue [21]. UALCAN is a fast and effective online analysis and mining website, mainly based on the TCGA database-related cancer data, and can provide a large number of comprehensive analyses, including gene expression, survival analysis, and epigenetic regulation [22].

### 2.5. Verification of Gene Expression in LAML Cohort II

Furthermore, an additional cohort of patients aged between 21 and 65 years was used for the identification of signaling pathways for the two selected genes, AP1G1 and SP1, as well as for all other genes significantly overrepresented in patients with longer and shorter survival periods. Samples were obtained from the GDC repository (https://portal.gdc.cancer.gov/, accessed on 7 May 2024). Raw RNAseq counts from peripheral blood samples of patients aged 21 to 65 were collected from the TCGA-LAML project. Patients with prior malignancy, treatment before sample collection, and a follow-up period of less than three years were excluded. This resulted in a final sample size of 49 patients. Raw gene counts for these patients were acquired, and differential gene expression (DGE) analysis was conducted. Samples from patients who survived longer than 3 years were compared with those who survived less than 3 years. Genes with low expression were excluded using the EdgeR [23] library in R. Raw counts were normalized to counts per million (CPM) and log-2-transformed using the voom function from the limma package in R. DGE analysis was performed using the limma package in R [24].

### 2.6. Survival Analysis of AP1G1 and SP1

We obtained the overall survival (OS) map data of AP1G1 and SP1 for LAML from the “Survival Map” module in GEPIA, using a 50% cutoff value to separate groups into high expression and low expression [21]. In addition, the UALCAN online tool was used to carry out a survival analysis of AP1G1 and SP1 [22]. In this study, the Kaplan–Meier (KM) plotter database was used to assess the overall survival of hub differentially expressed genes (DEGs) for two genes of LAML. KM plotter split the patient samples into two groups according to an automatically calculated optimal cutoff value to analyze the prognostic value of AP1G1 and SP1 in LAML and obtained the corresponding survival plots. *p* < 0.05 was accepted as the measure of the significance level.

### 2.7. Construction of Protein–Protein Interaction (PPI) Network

The STRING database is a protein–protein association network that provides all known and predicted information about the direct (physical) and indirect (functional) relationships that occur between different proteins [25]. Another online resource, GeneMANIA, is a database consisting of an intuitive interface for gene function predictions and interactions of genes with each other. The PPI network of AP1G1- and SP1-associated target genes was established by the STRING (http://www.string-db.org/, accessed on 7 May 2024), a search engine for interacting genes or proteins, and the GeneMANIA (http://genemania.org/, accessed on 7 May 2024) database [26]. Furthermore, a circRNA–miRNA–mRNA network was built by combining the derived circRNA–miRNA and miRNA–mRNA pairs.

### 2.8. Functions of AP1G1 and SP1

The CancerSEA (http://biocc.hrbmu.edu.cn/, accessed on 7 May 2024) tool was used to determine AP1G1 and SP1 functions in acute myeloid leukemia [27]. Additional DGE analyses were conducted (cohort II), comparing 25 samples with high SP1 and AP1G1 gene expression against 24 samples with low SP1 and AP1G1 expression. The results of the DGE analysis were then used for enrichment pathway analysis, which was performed using the ReactomePA package in R [28].

## 3. Results

### 3.1. Identification of CircRNAs in LAML

To investigate the molecular mechanisms promoting LAML progression, we first obtained information about circRNAs from the CircR2Disease database. Two out of six circRNAs are transcribed from the same gene, hsa_circ_0004277, and hsa_circ_0017446, so the input consisted of five circRNAs under gene symbols. In LAML, four of these circRNAs are upregulated, and two are downregulated (Table 1). Among them, hsa_circ_0058058, which has fewer studies in the literature, was selected for further investigation.

### 3.2. Identification of circRNA-Mediated miRNA in LAML

**Figure 1:**
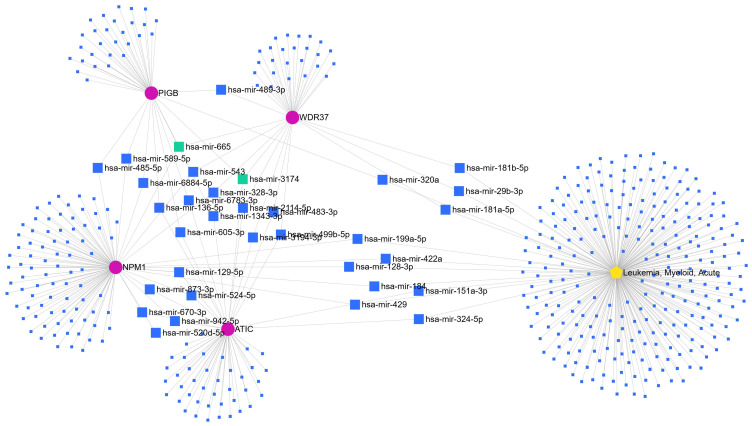
miRNA targets of NPM1, PIGB, ATIC, and WDR37 circRNAs based on miRNET network in LAML. Green squares represent miRNAs with three nodes and blue large squares represent miRNAs with two shared nodes between LAML (yellow pentagon shape) and circRNAs (pink circles).

Only four out of five listed circRNAs were mapped in miRNET interaction database, NPM1, PIGB, ATIC, and WDR37. miRNET software has found 464 miRNA nodes for one disease and 4 circRNAs. One miRNA, hsa-miR-3174, was recognized as the common target of three circRNAs, ATIC, PIGB, and WDR37, but not in LAML (without LAML node). One miRNA, hsa-miR-665, was recognized as a common target of three circRNAs, NPM1, PIGB, and WDR37. Thirty miRNAs share two nodes. Hsa-miR-422a, hsa-miR-199a-5p, hsa-miR-429, hsa-miR-184, and hsa-miR-128-3p were recognized as targets of NPM1 in LAML, hsa-miR-151a-3p and hsa-miR-324-5p as targets of ATIC in LAML, and hsa-miR-320a was recognized as a target of PIGB in LAML. Hsa-miR-29b-3p, hsa-miR-181b-5p, and hsa-miR-181a-5p were recognized as targets of WDR37 circRNA in LAML (Figure 1). Using Circular RNA Interactome software, we predicted potential target miRNAs for circATIC (hsa_circ_0058058), and one of them was hsa-miR-324-5p. The common hsa-miR-324-5p obtained from Circular RNA Interactome software and miRNET was selected.

### 3.3. Prediction of miRNA Targets

Using miRNET and miRDB, we predicted potential target mRNAs for miRNAs. The common target 20 genes obtained from miRNET and miRDB were determined (Table 2). To determine whether these 20 genes are associated with tumorigenesis, we first performed their expression analysis in LAML using the GEPIA database. Among these genes, we selected the upregulated AP1G1 and SP1 genes.

### 3.4. Verification of Gene Expression in LAML

**Figure 2:**
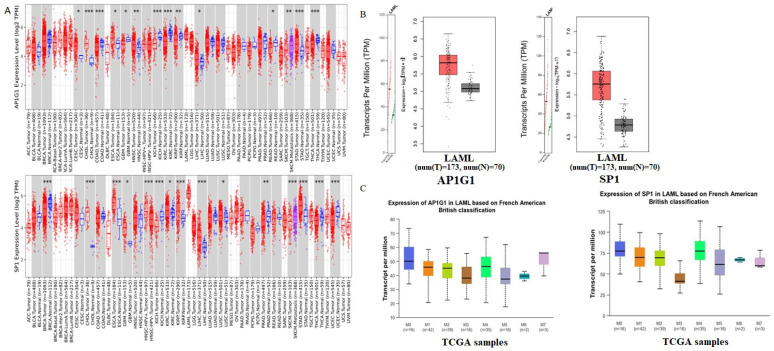
Differential expression of AP1G1 and SP1 in pan-cancer. (**A**) Expression levels of AP1G1 and SP1 in different TCGA tumors from TIMER database; * *p* < 0.05; ** *p*  <  0.01; *** *p*  <  0.001. (**B**) LAML in the TCGA database; matched TCGA normal and GTEx data were included as control. Box plot depicts AP1G1 and SP1 expression in tumor and normal tissues. * *p* < 0.05. (**C**) The expression of AP1G1 and SP1 in LAML analysis using the UALCAN database.

**Figure 3:**
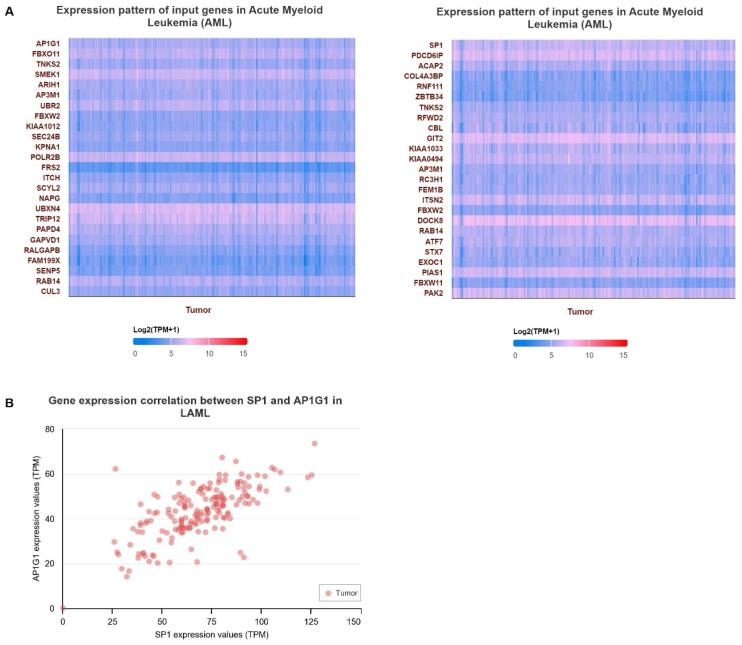
(**A**) Heatmaps of significant AP1G1 and SP1 in LAML. (**B**) The Pearson correlation analysis of AP1G1 and SP1 genes in LAML.

To examine the gene expression of AP1G1 and SP1, we first used the TIMER database to analyze the levels of AP1G1 and SP1 in different cancers (Figure 2A). AP1G1 and SP1 expression levels were upregulated in different types of human cancer (Figure 2A). We also examined the expression of AP1G1 and SP1 in LAML in the GEPIA and UALCAN databases, as shown in Figure 2B,C. After normal tissues from TCGA and GTEx databases were matched, higher AP1G1 and SP1 gene expression levels were observed in acute myeloid leukemia (LAML) (*p* < 0.05) compared to normal tissues (Figure 2B). There was a significant positive correlation between AP1G1 and SP1 gene pairs in LAML according to the Pearson-CC (R = 0.67) analysis from the UALCAN database. (Figure 3B). Moreover, a heatmap image of AP1G1, SP1, and related genes in LAML is shown in Figure 3A.

### 3.5. Survival Analysis of AP1G1 and SP1

**Figure 4:**
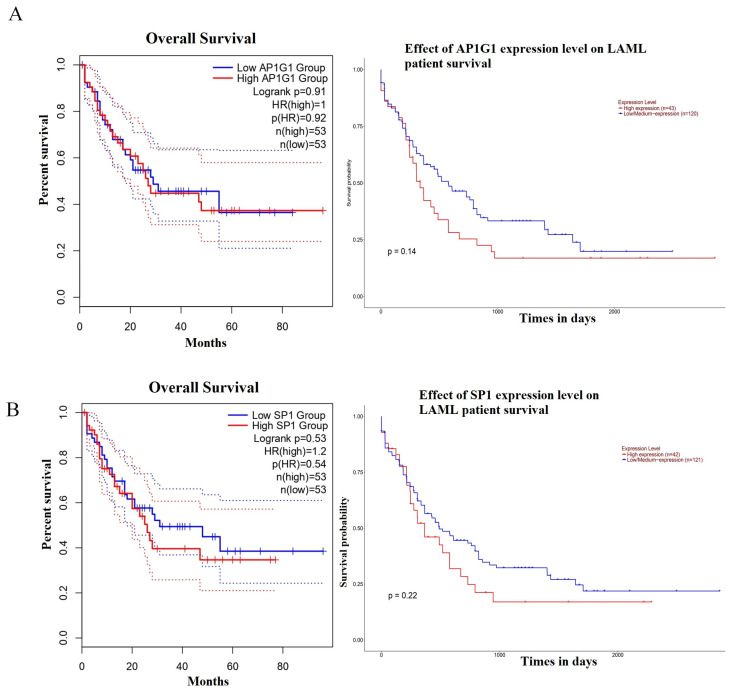
Analysis of the overall survival for AP1G1 and SP1 in LAML using the GEPIA and ULACAN databases: (**A**) AP1G1, (**B**) SP1.

The prognostic value of two-hub LAML was assessed using the Kaplan–Meier plotter database. The results showed that two hub DEGs (AP1G1 and SP1) had a low association with poor prognosis in LAML patients (*p* > 0.05) (Figure 4A,B). As shown in Figure 4A, LAML patients were associated with high expression of AP1G1 but had no significant prognosis (*p* = 0.14). In addition, a high expression level of SP1 was associated with poor prognosis for patients with LAML. However, SP1 hub DEGs had non-significant log-rank *p* values in LAML patients. (*p* = 0.22) (Figure 4B). The above results showed that the expression levels of AP1G1 and SP1 were observably positively correlated with short median survival and a poor prognosis for LAML.

### 3.6. Construction of Protein–Protein Interaction (PPI) Network

**Figure 5:**
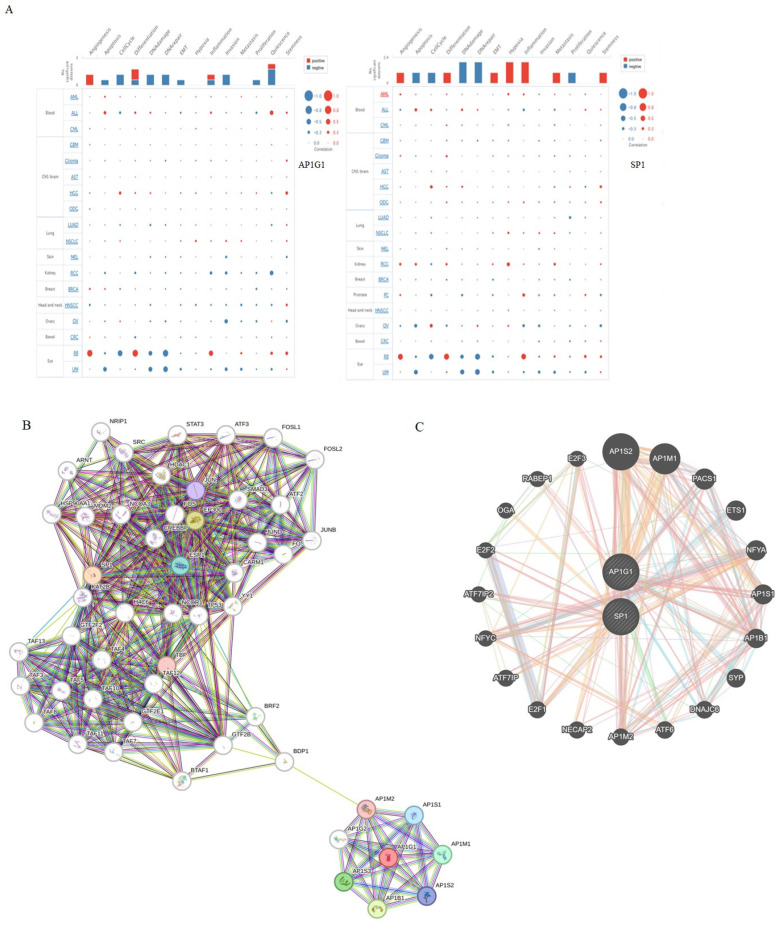
Analysis of the functions for AP1G1 and SP1 in LAML. (**A**) The functions of AP1G1 and SP1 in LAML analysis using the CancerSEA database. (**B**) The STRING database was employed to construct the protein interaction network of AP1G1 and SP1. (**C**) The gene interaction network of AP1G1 and SP1 was constructed using GeneMania.

We used the GeneMANIA and STRING databases to construct gene interaction and protein interaction networks with AP1G1 and SP1 (Figure 5B,C). The genes most closely associated with AP1G1 and SP1 were AP1S2, AP1M1, PACS1, AP1B1, AP1S1, E2F3, ATF7IP2, E2F2, ATF7IP, E2F1, ATF6, PACS1, ETS1, NFYA, SYP, DNAJC6, AP1M2, NECAP2, NFYC, OGA, and RABEP1 (Figure 5C). The proteins most closely associated with AP1G1 were AP1S2, AP1M1, AP1B1, AP1S1, and BDP1, and those most closely associate with SP1 were TP53, E2F1, ESR2, CREBBP, ESR1, and SMAD3 (Figure 5B).

### 3.7. Functions of AP1G1 and SP1

**Figure 6:**
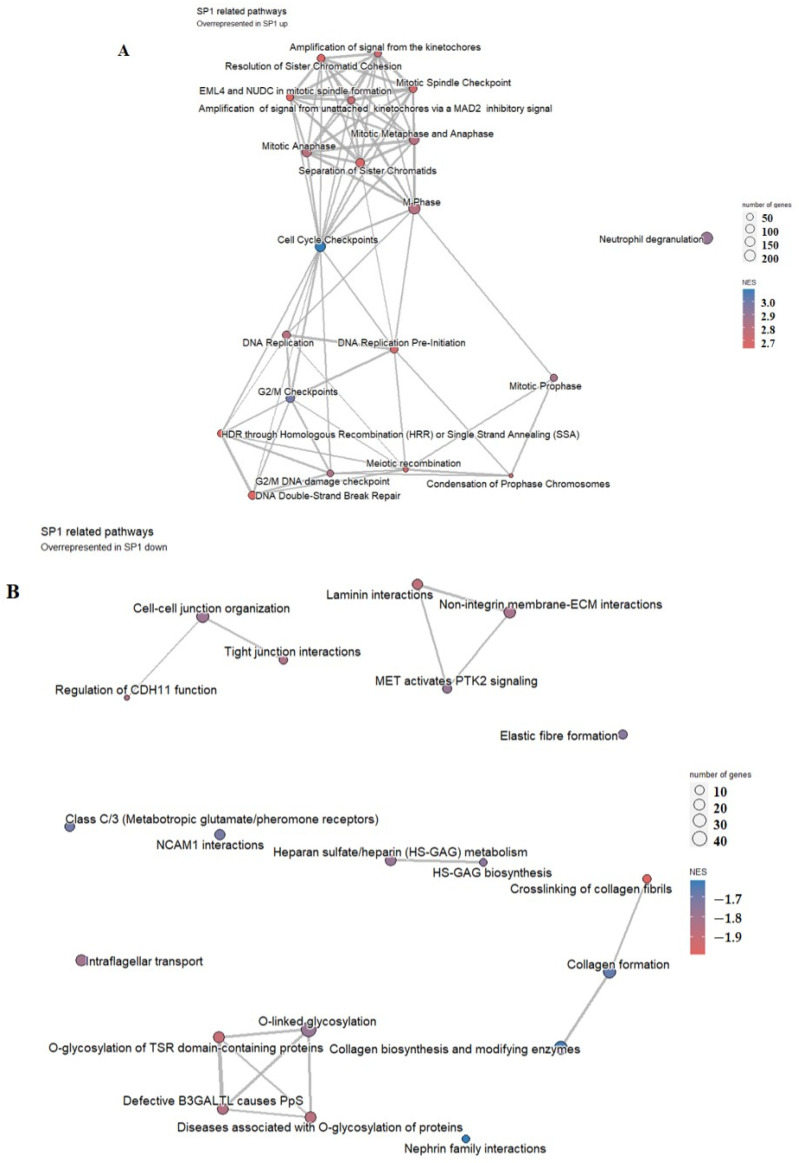
Pathway analysis of the group of 25 patients with higher (**A**) versus the group of 24 patients with lower (**B**) SP1 representation. Highly enriched pathways are represented by a red-to-blue spectrum, where the highest NES score is in the darkest blue.

**Figure 7:**
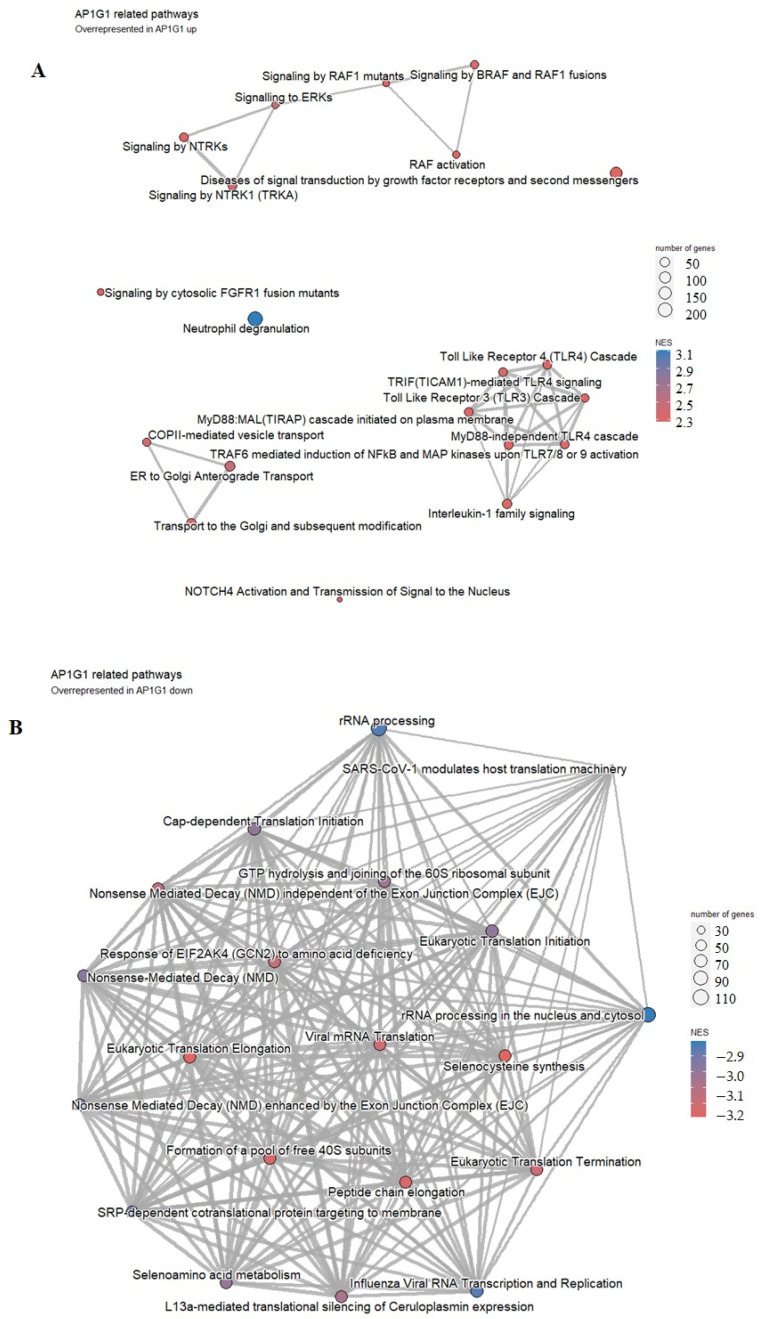
Pathway analysis of the group of 25 patients with higher (**A**) versus the group of 24 patients with lower (**B**) AP1G1 representation. Highly enriched pathways are represented by a red-to-blue spectrum, where the highest NES score is in the darkest blue.

We explored AP1G1 and SP1 functions in LAML using CancerSEA (http://biocc.hrbmu.edu.cn/, accessed on 7 May 2024). AP1G1 was involved mainly in angiogenesis, cell differentiation, inflammation, and quiescence in LAML (Figure 5A). SP1 was involved mainly in angiogenesis, cell differentiation, epithelial–mesenchymal transition (EMT), hypoxia, inflammation, metastasis, and stemness in LAML (Figure 5A). Additionally, functions of SP1 and AP1G1 (25 samples with higher SP1 and AP1G1 gene expression against 24 samples with lower SP1 and AP1G1 expression) were also investigated by enrichment pathway analysis with the ReactomePA package and are shown in Figure 6 and Figure 7.

In the group of patients with higher SP1 expression, pathway analysis has shown that highly enriched pathways were associated with cell division/cell cycle/DNA replication processes, homologous recombination, and DNA double-strand repair mechanisms (Figure 6A), while in the group with lower SP1 expression, overrepresented pathways were mostly associated with cell–cell adhesion, laminin and extracellular matrix interaction, and heparin metabolism, among others, as shown in Figure 6B.

In the group with higher AP1G1 expression, highly overrepresented pathways are neutrophil degradation, RAF1 and BRAF signaling pathways, Toll-Like Receptor 4 Cascade, and TRAF6 mediated induction of NfkB and MAP kinase pathways, among others (Figure 7A), while lower AP1G1 expression was associated with the processes of translation and rRNA processing, among other overrepresented pathways shown in Figure 7B.

### 3.8. DGE-LAML Analysis

The raw data from the TCGA_LAML project were divided into two groups, with survival longer than 3 years in one (“longer survival” group) and survival shorter than 3 years (“shorter survival” group) in the other, and were compared for all genes existing in the database. The data from patients with low follow-up who are still alive were excluded, as were all data from patients with previous malignancies and those who received therapy before sampling. In total, 49 samples (28 with shorter and 21 with longer survival) were analyzed.

**Figure 8:**
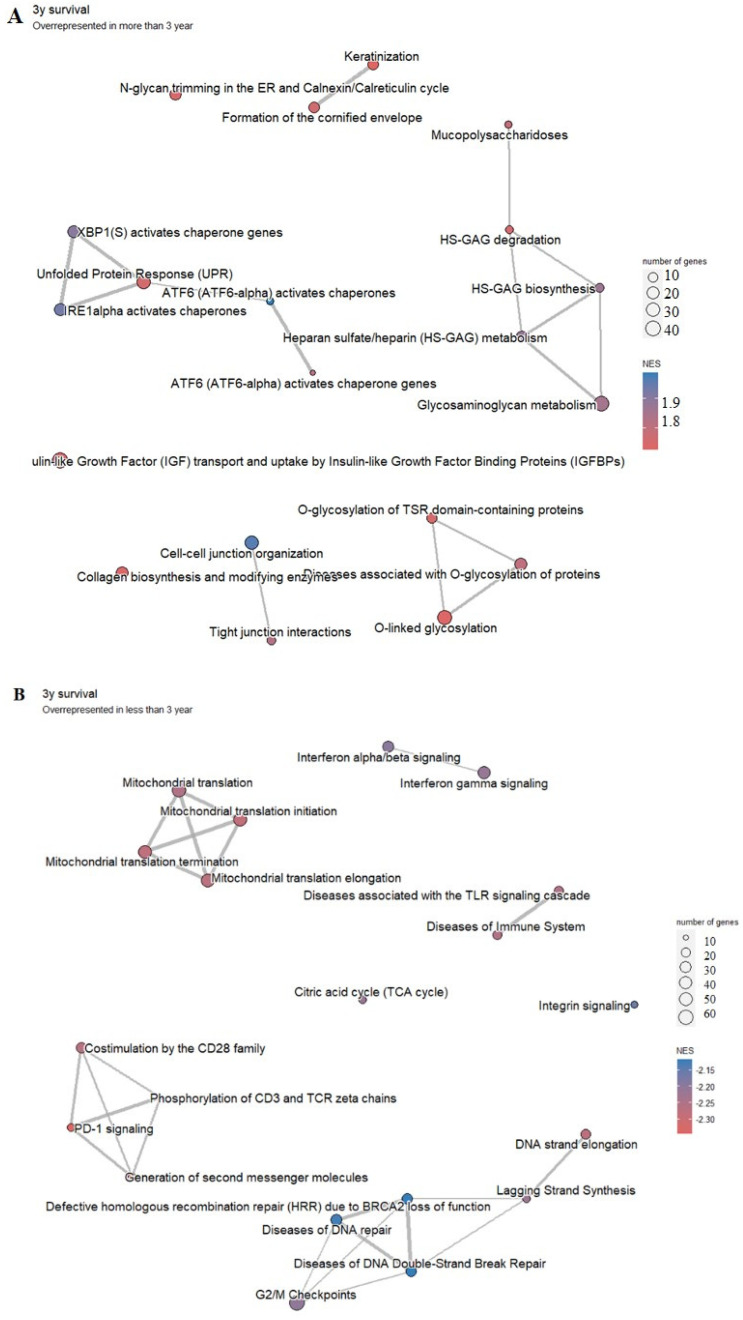
Pathway analysis of significantly overrepresented genes in LAML in the group of patients with longer survival (**A**) and with shorter survival (**B**). Highly enriched pathways are represented by a red-to-blue spectrum, where the highest NES score is in the darkest blue.

According to DGE analysis, 345 differential expressed genes (168 in the “longer survival” group and 177 in the “shorter survival” group, presented in a Supplementary Table) have been found. As for signaling pathways, there are a total of 266 significant processes and pathways (31 in the “longer survival” group and 235 in the “shorter survival” group, Figure 8A,B, respectively).

Among significantly overexpressed genes were poly(ADP-ribose) polymerase 1 (*PARP1*), nuclear factor kappa B subunit 1 (*NFKB1*), WD repeat domain 49 (*WDR49*), matrix metallopeptidase 7 (*MMP7*), and the gene for microRNA miR-6774 in the shorter survival group, while in the group with longer survival, significantly overrepresented genes included the mir-100-let-7a-2-mir-125b-1 cluster host gene, with the highest logFC (logarithm of fold change), 4.58, and the lowest adjusted *p*-value (0.018).

In the group of patients with better survival (more than 3 years), pathways associated with heparin metabolism and glycosylation are overrepresented (Figure 8A). In the group of patients with poorer survival (less than 3 years), PD-1 signaling may be one of the most affected related pathways, along with the signaling of defective homologous recombination repair (BRCA2 loss of function) and signaling associated with diseases of DNA repair, diseases associated with TLR signaling cascade, immune system diseases, and mitochondrial translation (Figure 8B).

## 4. Discussion

In recent years, while targeted approaches have shown promise in the fight against cancer, the treatability and prognosis of patients remain inadequate due to the shortage of drugs. Revealing the relationship of genes or molecules with the pathology and development of cancer is very important for new treatment methods [29]. The molecular mechanisms underlying the tumorigenesis and development of LAML are a relatively complex process due to the involvement of abnormalities in gene expression regulatory networks [30,31,32]. CircRNAs, as a type of stable, abundant, highly conserved, and tissue-specific regulatory RNA, have been shown to have a major impact on a wide range of biological processes. Moreover, the tumorigenesis and response to treatment of hematological malignancies are closely linked to the dysregulation of circRNAs [33]. Recent research indicates that aberrantly produced circRNAs may play a major role in controlling the development of LAML through various mechanisms [30,31,32].

In this study, we discovered a putative circRNA–miRNA–mRNA regulation network implicated in the pathophysiology of LAML using a series of database analyses. We identified six circRNAs using database data; two of these circRNAs being downregulated and four being upregulated, and selected circ-ATIC and hsa_circ_0058058 for further investigation. Abnormal ATIC, as the host gene of circ-ATIC, has also been implicated in the development of many types of cancer such as hepatocellular cancer [34], multiple myeloma tissues [35], and lung adenocarcinoma [36]. The interaction between circRNAs and their host genes is largely unknown. It has been stated that circ-ATIC does not affect the mRNA and protein level of ATIC, indicating that circ-ATIC functions by targeting miRNAs [37]. Li et al. showed that three circRNAs (hsa_circ_0035381, hsa_circ_0004136, and hsa_circ_0058058) were upregulated and two circRNAs (hsa_circ_0017446 and hsa_circ_0004277) were downregulated in acute myeloid leukemia patients [38].

It has been shown that circRNAs can bind to miRNAs as RNA sponges and increase or decrease gene expression by regulating miRNA activities and inhibiting or contributing to tumor development [9]. Therefore, we predicted the potential miRNAs targeted by circRNA. Bioinformatic analysis was performed using mirNET and Circ interactome software to search for candidate miRNA targets of circ-ATIC. As shown in Figure 1, we identified the possible target of circ-ATIC, hsa-miR-324-5p, by both mirNET and Circ interactome databases.

miR-324 has important implications in the pathogenesis of human diseases. Gene ontology studies have indicated that miR-324 has a role in various processes, such as the responses of cells to anti-leukemia factor, long-term synaptic strengthening, and positive regulation of cytokine production [39]. miR-324-5p has been reported to have antitumor effects in many cancers, including MM [40,41]. According to Gürel’s study, which used high-throughput expression data, hsa-miR-324-5p expression declined in LAML [11].

Bhise et al. showed a negative association between miR-324-5p with apoptotic rates of LAML cells induced by cytarabine treatment of eight LAML cells, but not with cytotoxicity measured by MTT assay [40]. Additionally, circulating miR-324-5p (among other 68 miRNAs) is differentially expressed (significantly upregulated) in bone marrow patients with a new LAML diagnosis compared with patients in complete remission (three months after treatment), where it was significantly reduced after treatment [2]. According to Leoncini et al.’s findings, higher levels of miR-324-5p were associated with LAML relapse. Furthermore, miR-324-5p was around 2.5 down-represented in patients with LAML who never relapsed compared with healthy individuals [42].

We predicted potential miRNA-targeted mRNAs via miRNET and miRDB, and after intersecting the results, we performed a series of analyses. We identified two important targets of miR-324-5p, AP1G1 and SP1 (Table 2).

Adaptin proteins (APs) play a crucial role in intracellular trafficking. Adapter-associated protein complex 1 gamma 1 subunit (AP1G1) is a gamma-adaptin of the large subunit family of adapter complexes [43]. AP1G1 expression is increased in various types of cancer, including head and neck, colorectal, breast, and brain cancer [44].

Although the biological significance of increased AP1G1 expression in cancer is not yet clear, AP1G1 is known to play a critical role in early development [44,45]. Sp1 (Specificity protein 1) is a well-known member of the family of transcription factors involved in a wide variety of fundamental biological processes. SP1 has been proven to be important in cell growth, differentiation, apoptosis, cell death, and carcinogenesis [46,47]. Sp1 is a crucial transcription factor for numerous genes as a result. Abnormal Sp1 expression and activation are thought to promote the initiation and progression of human cancer, including leukemia [47]. SP1 is regulated by multiple miRNAs in various human cancers [46]. For example, it was shown that the miR-29b/Sp1/FUT4 axis promotes the malignant behavior of leukemia stem cells by regulating CD44 through the Wnt/β-catenin pathway [47].

Gene alterations are crucial to the development of tumors. Using the GEPIA database and UALCAN tool, expression analyses of AP1G1 and SP1 genes in LAML were performed. In the GEPIA analysis, with TCGA normal data and a database based on GTEx data in 173 LAML patients and 70 healthy subjects, AP1G1 and SP1 expression levels were higher in LAML patients (Figure 2B). Similarly, we found that AP1G1 and SP1 were significantly expressed in LAML using the UALCAN tool (Figure 2C).

The survival prognosis analysis of AP1G1 and SP1 indicated a consistent conclusion. The results of survival analysis with GEPIA tools showed that high expression levels of AP1G1 and SP1 were positively correlated with a poor prognosis concerning LAML. Moreover, AP1G1 and SP1 may be related to oncogenesis and proliferation in the UALCAN database based on the results of the gene expression analysis and survival analysis from GEPIA. Notably, AP1G1 and SP1 were highly expressed in LAML; however, high expression of AP1G1 and SP1 was low related to a poor prognosis for LAML. The above results of gene expression analysis and survival analysis indicate that AP1G1 and SP1 may be tumor-related factors significantly related to the genesis and progression of LAML. Indeed, these results demonstrated a correlation between prognosis and survival in LAML and alteration in the AP1G1 and SP1 genes. In addition, these results suggested that AP1G1 and SP1 might play different roles in LAML. Further experiments are necessary to explore whether AP1G1 and SP1 play a precise role in these cancer types and to clarify the mechanism.

Functional enrichment analysis and PPI network analysis were performed to identify the proteins that AP1G1 and SP1 are associated with in the development of LAML and to reveal how these proteins function. Our analysis revealed the biological background of those two genes and the potential association with differences in signaling cascades in the cases of down- or upregulated genes. To explore the molecular mechanism of LAML, the LAML-related PPI network was constructed, and 266 hub DEGs were identified. These hub DEGs were all overexpressed in significant processes and pathways in LAML. The Kaplan–Meier plotter was used to evaluate the effects of the two hub genes on the survival of LAML patients. The results showed that overexpression of AP1G1 and SP1 led to poor prognosis in LAML. As a main component, AP1G1 plays an important role in membrane protein sorting in endosomes after receptor-mediated endocytosis. The expression of AP1G1 was found to be increased in several types of cancers, including head and neck, colorectal, breast, and brain cancer. Studies have shown that the knockdown of AP1G1 reduces the level of the ASCT2-EGFR complex. As a result, it decreases intracellular glutamine uptake and glutathione biosynthesis [44]. Studies have indicated that SP1 expression is elevated in some cancer types, including glioblastoma [48], lung cancer [49], breast cancer [50], and cervical cancer [51], and that these conditions are linked to a poor prognosis. Additionally, SP1 has been shown to promote carcinogenesis and cause metabolic reprogramming in a variety of cancer types [51].

Studies have shown that AP1G1 and SP1 are more highly expressed in cancer tissues than in normal tissues, and both are related to the invasion and proliferation of cancer cells [44,51]. Our findings highlighted the possibility that PPI and hub DEGs are involved in the etiology and progression of LAML. In addition, the results indicated that 21 genes and 11 proteins most closely associated with AP1G1 and SP1 in the PPI network were distributed in the modules, suggesting that these genes may have important roles in LAML.

Moreover, pathway analysis revealed that PD-1 signaling may be a highly enriched pathway, along with the signaling of impaired homologous recombination repair components, the signaling associated with DNA-repair-related diseases, and the signaling associated with the TLR signaling cascade and immune response. PD-L1, a receptor for PD-1, is shown to be associated with acute myeloid leukemia via the activation of the PI3K-AKT pathway, apoptosis, and proliferation [52] and is marked as a potential target for the treatment of leukemia. The PD-1/PD-L1 axis and signaling may be responsible for the evasion of the immune response, thus highlighting PD-1/PD-L1 checkpoint inhibitors as potential targets for the treatment of LAML, which are still in different stages of clinical trials [53].

According to the findings above, it has been demonstrated that circRNAs can target miRNAs and miRNAs can modulate various cellular functions posttranscriptionally. We suggest that altered miRNA and circRNA expression using overexpressing plasmids or small interfering RNAs may have clinical potential for LAML treatment. Downregulation of hsa circ -0058058 may contribute to the anticancer functions of miR-324-5p on LAML tumorigenesis, and upregulation of miR-324-5p can abolish the oncogenic effects of AP1G1 and SP1 on LAML tumorigenesis. Also, miR-324-5p might be a chemosensitive miRNA, whose levels change after the treatment of LAML, both in vitro and in vivo. Higher miR-324 levels might be associated with worse outcomes and response to therapy of LAML patients and higher chances for the relapse of LAML. But, on the other hand, this miRNA was lower in patients with LAML who never experienced relapse, compared with healthy controls, indicating that different pathways may be activated in the formation and progression of LAML between patients who will differently respond to therapy. These findings may indicate that miR-324 may be better as a parameter for the prediction of LAML treatment (future prognostic and predictive biomarker) than as a diagnostic tool for LAML. This chemosensitive feature should be used in further investigations to monitor patients under the treatment of LAML on a weekly basis to define its profile and to give directions in the future on how to modify treatment to accomplish the highest efficiency and avoid unnecessary side effects, especially for highly sensitive patients whose leukemia does not retreat during the treatment.

This study has a few limitations. Only some databases were used as databases of miRNA profiles and circRNA are rare. Experimental validation was not performed for the biological functions and targets of the identified miRNA and circRNA. Further studies are needed for the mechanism of miR-324-5p and its potential targets in the development of LAML. We believe that it can be a potential biomarker for the early diagnosis of LAML patients and that cohort studies should be conducted on this topic.

## 5. Conclusions

In conclusion, we successfully established a miRNA–mRNA regulatory network mediated by hsa_circ_0058058 in LAML. This study utilized various bioinformatics tools to conduct a comprehensive analysis of the role of hsa_circ_0058058-regulated AP1G1 and SP1 expression in LAML and carried out some verification on this basis. This study first showed that hsa_circ_0058058 regulated AP1G1 and SP1 expression through miR-324-5p to promote angiogenesis, cell cycle, and DNA replication processes, suggesting a novel therapeutic target for LAML treatment. Although further experimental and clinical validation is required, targeted molecules within this network may potentially serve as diagnostic and therapeutic biomarkers for disease. This may provide new insights into the molecular mechanisms of LAML and potential therapeutic targets.

## Figures and Tables

**Table 1:** List of circRNAs and their genes in LAML.

CircRNA ID	Location	Gene	Expression Levels
hsa_circ_0075001, circNPM1	chr5:170817054-170827214	NPM1	Up
hsa_circ_0004277	chr10:1125950-1126416	WDR37	Down
hsa_circ_0035381	chr15:55621921-55634000	PIGB	Up
hsa_circ_0004136	chr6:73713630-73751785	KCNQ5	Up
hsa_circ_0058058	chr2:216177220-216190861	ATIC	Up
hsa_circ_0017446	chr10:1125950-1132297	WDR37	Down

**Table 2:** Prediction of miRNA targets from miRNET and miRDB.

AP1G1	RBL1	ERLIN2	DNAJC11	SMARCA4
VDAC1	PIGM	RAN	CUEDC2	PAFAH1B1
ELAVL1	UBE2I	SP1	SLC16A1	SETD5
THAP11	STRN	CHTOP	ARF3	HTT

## Data Availability

The data presented in this study are available on request from the corresponding author.

## Supplementary Materials

The following supporting information can be downloaded at: https://www.mdpi.com/article/10.3390/biology13070487/s1, Table S1: DGE list.
